# Clinical and immunological data of nine patients with chronic mucocutaneous candidiasis disease

**DOI:** 10.1016/j.dib.2016.02.040

**Published:** 2016-02-23

**Authors:** Laura Dotta, Omar Scomodon, Rita Padoan, Silviana Timpano, Alessandro Plebani, Annarosa Soresina, Vassilios Lougaris, Daniela Concolino, Angela Nicoletti, Giuliana Giardino, Amelia Licari, Gianluigi Marseglia, Claudio Pignata, Nicola Tamassia, Fabio Facchetti, Donatella Vairo, Raffaele Badolato

**Affiliations:** a Department of Clinical and Experimental Sciences, Institute of Molecular Medicine “Angelo Nocivelli”, University of Brescia, Brescia, Italy; b Unit of Paediatric Pneumonology, Spedali Civili of Brescia, Brescia, Italy; c Department of Paediatrics, University of Catanzaro, Catanzaro, Italy; d Department of Translational Medical Sciences, Federico II University, Naples, Italy; e Department of Paediatrics, Foundation IRCCS Policlinico San Matteo, University of Pavia, Pavia, Italy; f Department of Medicine, General Pathology Unit, University of Verona, Verona, Italy; g Department of Molecular and Translational Medicine, Pathology Unit, University of Brescia, Brescia, Italy; h Department of Molecular and Translational Medicine, University of Brescia, Brescia, Italy

## Abstract

This paper describes the heterogeneous clinical phenotype of a cohort of nine patients diagnosed with heterozygous mutations in *STAT1*. We report data of extended immunophenotyping over time and we show lung damage in four patients. The increased phosphorylation of STAT1 in response to IFNγ and IFNα stimulation proves the gain-of-function nature of the defects. The data are supplemental to our original article concurrently published “Clinical heterogeneity of dominant chronic mucocutaneous candidiasis disease: presenting as treatment-resistant candidiasis and chronic lung disease” [1], where additional results and interpretation of our research can be found.

**Specifications Table**

**Table:** 

Subject area	Medicine
More specific subject area	Immunology, Primary immunodeficiencies
Type of data	Text file, table and figures
How data was acquired	Survey, computed tomography reading, flow cytometry (BD Phosflow), FACSCalibur (BD Bioscence), FlowJo version 7.5 Software (TreeStar)
Data format	Raw and analyzed
Experimental factors	Peripheral blood was treated with IFNγ or IFNα that modulate STAT1 phosphorylation
Experimental features	Lymphocytes and monocytes were isolated from peripheral blood and processed with specific antibody for evaluation of phosphorylated STAT1.
Lymphocytes were analyzed in their different subsets
Data source location	Department of Clinical and Experimental Sciences, Institute of Molecular Medicine “Angelo Nocivelli”, University of Brescia, Brescia, Italy
Department of Paediatrics, Spedali Civili of Brescia, Brescia, Italy
Department of Paediatrics, University of Catanzaro, Catanzaro, Italy
Department of Translational Medical Sciences, Federico II University, Naples, Italy
Department of Paediatrics, Foundation IRCCS Policlinico San Matteo, University of Pavia, Pavia, Italy
Data accessibility	All data are provided in this article

**Value of the data**

• The data provide novel clinical and immunological phenotypes of the emerging highly heterogeneous primary immunodeficiency due to heterozygous mutations of *STAT1*.
• The data of the increased STAT1 phosphorylation in response to IFNs confirm the gain-of-function as the pathogenetic mechanism of dominant mutations in *STAT1*.
• The data of immunophenotyping may stimulate further research on the molecular pathways leading to lymphopenia and highlight the clinical utility of monitoring of lymphocyte subsets as prognostic marker.

## 1 Data

We describe in details the clinical phenotype of a cohort of nine pediatric or adult patients who mainly presented with chronic mucocutaneous candidiasis disease (CMCD) and were consequently screened for *STAT1* mutations [1]. After identification of heterozygous defects, the gain-of-function nature of the *STAT1* variants was demonstrated by prolonged phosphorylation of STAT1 with exaggerated signaling through IFNγ and IFNα pathways for both previously reported (Fig. S1) [2], [3], [4] and for novel variants [1]. The heterogeneous clinical phenotype was analyzed in term of severity of CMCD, type of infections, autoimmune manifestations, detailed immunophenotyping over time (as detailed in Table S1), and long-term complications, particularly developing of chronic lung disease (as illustrated in Fig. S2).

## 2 Experimental design, materials and methods

### 2.1 Clinical survey

Medical history and clinical data were compiled retrospectively and prospectively from medical records of all patients of our cohort. Patients who were treated primarily at the Departments of Brescia were selected for pulmonary evaluation and underwent pulmonary function tests and computed tomography imaging to stage lung disease. Extended immunophenotyping was performed. All patients or their guardians provided written informed consent under approved protocols of their center of reference. All patients were Caucasian and their clinical phenotype is herein detailed.

#### 2.1.1 Patient 1

The patient is a 11-year-old boy born to nonconsanguineous parents. At the age of 1 year he began to experience recurrent oral thrush. He once developed skin candidiasis (forehead), and since the age of 8 years he has persistent onychomycosis of his second hand finger. Oropharyngeal and skin candidiasis responded first to oral fluconazole, but requiring oral itraconazole (100 mg/die) following resistance. Nail infection never responded to these medications. Since early infancy he suffered from recurrent upper respiratory tract infections, he had one episode of right pneumonia at the age of 5 years, and several bouts of obstructive bronchitis. At the age of 6.5 years blood tests revealed increased TSH (7.155 mU/L, range 0.27–4.2), and he was commenced on levothyroxine at the age of 9 years (following increase of TSH to 61.63 mU/L). Thyroid antibodies tested negative, but thyroid ultrasound showed dishomogeneous appearance of the gland with hypoechoic areas. His immunological assessment revealed low IgM (31 mg/dl, range>49) on repeated determinations since the age of 6 years.

#### 2.1.2 Patient 2

Mother of patient 1, this 45-year-old woman presented, since early childhood, with severe oropharyngeal and nails chronic candidiasis, resistant to oral antifungal treatment (first fluconazole and voriconazole, then itraconazole progressively increased from 100 to 400 mg/die). Since her adolescent age, she suffered from oesophageal candidiasis, requiring intravenous antifungal treatment (caspofungin). She suffered from recurrent skin boils, and she experienced episodes of recurrent fever associated to weight loss. She had bouts of pulmonary infections and a chest computed tomography (CT)-scan performed when she was 21 year-old showed bronchiectasis in the left lower lobe and the lingula (Fig. S2). She developed recurrent infection with *Pseudomonas aeruginosa* at the age of 31 year-old, causing higher frequency of pulmonary exacerbations. She had gestational diabetes, but never developed autoimmune manifestations. In her adulthood, she developed persistent T and B cells lymphopenia (Table S1), with unprotective antibody response to vaccine (anti-tetanus toxoid antibody 0.02 UI/ml).

#### 2.1.3 Patient 3

A 33-year-old man was born to nonconsanguineous parents. He presented with recurrent oral thrush since the age of 6 months, well responding to oral treatment (first fluconazole then itraconazole, progressively increased from 100 to 200 mg/die). He was diagnosed with oesophageal candidiasis at the age of 19 years, well responding to intravenous treatment (amphotericin B). Since childhood, he had repeated episodes of otitis media and multiple bouts of pneumonia. Bronchiectasis were firstly documented at the age of 10 years in the left lower lobe and the lingua (Fig. S2). He also suffered from recurrent skin boils. At the age of 15 years he presented with multiple granulomatous necrotizing lymphadenitis caused by *Cryptococcus neoformans* infection, which responded to intravenous fluconazole. At the age of 28 years he developed visceral leishmaniasis, was treated with intravenous amphotericin B, but, because of drug-related nephropathy, was replaced with miltefosine. He also had recurrent molluscum and Human Papilloma Virus (HPV) (warts) infections. He developed hypothyroidism at the age of 32 years (TSH>100 mU/L), associated to negative thyroid antibodies, and was started on daily levothyroxine. On autoimmunity screening, he has positive antinuclear antibodies (title 1:320) and positive anti-double stranded DNA antibody (138–122%, normal value <35%), clinically associated to systemic lupus erythematosus (SLE)-like facial skin lesions. Immunological investigations detected poor vaccine response to tetanus toxoid (0.07 UI/ml), associated to persistent T cells lymphopenia and NK cells defect by his adult age (Table S1).

#### 2.1.4 Patient 4

A 15-year-old boy was born at term to nonconsanguineous parents. Since childhood, he presented with recurrent respiratory tract infections complicated with chronic sinusitis. At the age of 5 years he first had oral thrush secondary to antibiotic therapy, and since then oral candidiasis occurred when on antibiotics, easily responding to topical antifungal (miconazole). At the age of 14 years he developed bone marrow aplasia requiring platelets and blood transfusions, likely cotrimoxazole-related, as he had been started on antibiotic prophylaxis for respiratory infections. On that occasion, a chest CT-scan was done, revealing bronchiectasis and thickening of bronchial walls bilaterally in the lower lobes and the lingua, and the right middle lobe (Fig. S2). During the following year, he experienced two pulmonary exacerbations caused by *Serratia marcescens*, and one due to *Pseudomonas aeruginosa*, the latter eradicated following oral ciprofloxacin treatment. He has vitiligo and positive antinuclear antibodies (title 1:320). He has poor antibody response (anti-tetanus toxoid antibody 0.03 UI/ml); a determination of low CD4+T cells was detected once (Table S1).

#### 2.1.5 Patient 5

A 10-year-old boy was born at term to nonconsanguineous parents. From 2 months of age he had chronic oral thrush and suffered from gastroesophageal reflux. At the age of 4 months he had one episode of severe bilateral pneumonia with *Pneumocystis jirovecii* (PCC), complicated by *Streptococcus viridans* and *Candida albicans* sepsis. Lymphopenia was detected in his first year of life (Table S1). He subsequently suffered from multiple bouts of pneumonia, but he is currently free of bronchiectasis. When he was 4-year-old an endoscopy showed esophageal Candida infection, and he was treated with intravenous voriconazole with transitory benefit. A severe gastroesophageal reflux disease led to a Nissen fundoplication at the age of 5 years. Unfortunately he had a fundoplication leak with secondary hiatal hernia and poor feeding, and *Candida* infection recurred. He responded to oral itraconazole (200 mg/die). He presents short stature, but growth hormone deficiency was ruled out.

#### 2.1.6 Patient 6

A 7-year-old girl was born at term to nonconsanguineous parents. She presented with chronic candidiasis of oral mucosa, head skin, and hand nails from the age of 2 years, responding to oral treatment (fluconazole 50 mg/die). She had several episodes of respiratory tract infections, including pneumonia with *Pseudomonas aeruginosa*. A CT-scan at the age of 6 years showed bronchiectasis in the right middle lobe and the lingula (Fig. S2). She has microcytic anemia and was started with iron therapy.

#### 2.1.7 Patient 7

A 14-year-old girl was born at term to nonconsanguineous parents. Since the age of 6 months she suffered from vaginal candidiasis, responding to topical antifungal treatment, and recurrent oropharyngeal candidiasis (treated with fluconazole from 100 to 200 mg/die). She also suffered from chronic sinusitis and one episode of pneumonia was recorded at the age of 11 years.

#### 2.1.8 Patient 8

A 17-year-old boy was born preterm (36 weeks) to nonconsanguineous parents. Since he was 7 year-old he presented with recurrent oropharyngeal and nails candidiasis, followed by onset of oesophageal localization in his adolescent age, well responding to oral fluconazole. At the age of 8 years he suffered from severe chicken pox, and since he was 11 year-old he experienced recurrent herpetic infections involving genitals and limbs, recurrent *S. aureus* skin abscesses and suppurative eyelid infections. He also developed parodontitis.

#### 2.1.9 Patient 9

A 8-year-old girl was born at term to nonconsanguineous parents. She presented with recurrent oral thrush since the age of 5 months, associated with oesophageal *Candida* colonization, and followed by nail candidiasis by 1 year of age. Mucocutaneous candidiasis responded to oral fluconazole. She had one episode of lobar pneumonia at the age of 7 months and recurrent upper respiratory tract infections. Since she was 2 year-old she suffered from recurrent infections with Herpes Simplex Virus (episcleritis) and Varicella-Zoster Virus (disseminated skin lesions). She developed hypothyroidism (TSH 100 mU/L, but negative thyroid auto-antibodies) at the age of 7 years, and was started on daily levothyroxine treatment.

### 2.2 Flow cytometry

Written informed consent was obtained from patients or parents (for minors) and healthy control to collect blood for *in vitro* functional studies. Peripheral blood from both patients and healthy donors was left unstimulated and stimulated with IFNγ (1000 U/ml), or IFNα (40,000 U/ml), for 30 min. Cells were lysed, permeabilized, and stained, as indicated by the manufacturer (BD Phosflow). Specific phycoerythrin-labeled antibody for phosphorylated STAT1 (pSTAT1) (pY701; BD Biosciences) was then used, and pSTAT1 was evaluated in both lymphocyte and monocyte gates. Cells were acquired using FACSCalibur (BD Bioscence) and analyzed by FlowJo version 7.5 Software (TreeStar).

### 2.3 Statistical analysis

Statistical analyses were performed using GraphPad Prism Version 5.0 (GraphPad Software, San Diego, CA). Statistically significant differences were assessed by nonparametric two-side Mann–Whitney *U*-test with 95% confidence bounds. Significance was accepted at *P*<0.05.

## Appendix A Supplementary material

Fig 1 Supplementary material . Intracellular staining of phosphorylated tyrosine 701 STAT1 (P701-YSTAT1) in not stimulated and IFNα-induced T lymphocytes (A), and not stimulated and IFNγ-induced monocytes (B). NS=Not Stimulated; HD=Healthy Donor. Fig 2. Chest Computed Tomography scans showing diffuse bronchiectasis in four GOF-STAT1 patients developing progressing chronic lung disease.
