# Supplementary material for: Clinical and immunological data of nine patients with chronic mucocutaneous candidiasis disease
Source: Data Brief. 2016 Feb 23;7:311–5. doi: 10.1016/j.dib.2016.02.040 (PMC4777981; doi:10.1016/j.dib.2016.02.040)
Supplement: Fig 1 — Supplementary material . Intracellular staining of phosphorylated tyrosine 701 STAT1 (P701-YSTAT1) in not stimulated and IFNα-induced T lymphocytes (A), and not stimulated and IFNγ-induced monocytes (B). NS=Not Stimulated; HD=Healthy Donor. Fig 2. Chest Computed Tomography scans showing diffuse bronchiectasis in four GOF-STAT1 patients developing progressing chronic lung disease. [file mmc1.zip › Supplementary Table 1.docx]

**Table 1. Lymphocyte subset variations over time of CMCD patients showing lymphopenia by adulthood and reduction of memory B cells.**

|  |  | **Age 6-12mo** | **Age 2-5 yr** | **Age 5-10 yr** | **Age 10-18 yr** | **Age >18 yr** |
| --- | --- | --- | --- | --- | --- | --- |
| **P1** | Total Lymphocytes |  | 2810 | 1330 | 2100 |  |
|  | CD3^+^ T cells |  | 1767 | 840 | 1514 |  |
|  | CD3^+^CD4^+^ T cells |  | 1115 | 365 | 611 |  |
|  | CD3^+^CD8^+^ T cells |  | 473 | 227 | 459 |  |
|  | CD16^+^ NK cells |  | **100** | 292 | 226 |  |
|  | CD19^+^ B cells |  | 826 | 273 | 424 |  |
|  | Naïve CD19^+^CD27^-^ cells |  |  |  | 59.4% of CD19^+^ (33-60.7) |  |
|  | IgM memory CD27^+^IgM^+^ cells |  |  |  | **3.6**% of CD19^+^ (5.3-22.9) |  |
|  | Switched memory CD27^+^IgM^-^ cells |  |  |  | **1.4**% of CD19^+^ (2.5-16.3) |  |
| **P2** | Total Lymphocytes |  |  |  |  | **810** |
|  | CD3^+^ T cells |  |  |  |  | **699** |
|  | CD3^+^CD4^+^ T cells |  |  |  |  | 421 |
|  | CD3^+^CD8^+^ T cells |  |  |  |  | 128 |
|  | CD16^+^ NK cells |  |  |  |  | 160 |
|  | CD19^+^ B cells |  |  |  |  | **49** |
| **P3** | Total Lymphocytes |  |  |  | 1989 | **710** |
|  | CD3^+^ T cells |  |  |  | **1217** | **375** |
|  | CD3^+^CD4^+^ T cells |  |  |  | 664 | **233** |
|  | CD3^+^CD8^+^ T cells |  |  |  | 485 | 128 |
|  | CD16^+^ NK cells |  |  |  | 131 | **13** |
|  | CD19^+^ B cells |  |  |  | 710 | 261 |
|  | Naïve CD19^+^CD27^-^ cells |  |  |  |  | 78.9 of CD19^+^ (29-70) |
|  | IgM memory CD27^+^IgM^+^ cells |  |  |  |  | **1** of CD19^+^ (8.2-30) |
|  | Switched memory CD27^+^IgM^-^ cells |  |  |  |  | **1.4** of CD19^+^ (7.2-26.3) |
| **P4** | Total Lymphocytes |  |  | 1780 | 1510 |  |
|  | CD3^+^ T cells |  |  | 1172 | 1402 |  |
|  | CD3^+^CD4^+^ T cells |  |  | 416 | **382** |  |
|  | CD3^+^CD8^+^ T cells |  |  | 666 | 916 |  |
|  | CD16^+^ NK cells |  |  | 204 | 122 |  |
|  | CD19^+^ B cells |  |  | 236 | 102 |  |
|  | Naïve CD19^+^CD27^-^ cells |  |  |  | 78.5 of CD19^+^ (33.8-79.6) |  |
|  | IgM memory CD27^+^IgM^+^ cells |  |  |  | 5.5 of CD19^+^ (3.5-24.1) |  |
|  | Switched memory CD27^+^IgM^-^ cells |  |  |  | **0.4** of CD19^+^ (2.7-20.6) |  |
| **P5** | Total Lymphocytes | **3570** | 2730 | 1950 |  |  |
|  | CD3^+^ T cells | 2084 | 1850 | 1599 |  |  |
|  | CD3^+^CD4^+^ T cells | **1153** | 895 | 877 |  |  |
|  | CD3^+^CD8^+^ T cells | 788 | 679 | 565 |  |  |
|  | CD16^+^ NK cells | 147 | **74** | **74** |  |  |
|  | CD19^+^ B cells | 1245 | 717 | 260 |  |  |
|  | Naïve CD19^+^CD27^-^ cells |  |  | 71.2 of CD19^+^ (33-60.7) |  |  |
|  | IgM memory CD27^+^IgM^+^ cells |  |  | **1.9** of CD19^+^ (5.3-22.9) |  |  |
|  | Switched memory CD27^+^IgM^-^ cells |  |  | **0.6** of CD19^+^ (2.5-16.3) |  |  |
| **P6** | Total Lymphocytes |  |  | 2910 |  |  |
|  | CD3^+^ T cells |  |  | 1316 |  |  |
|  | CD3^+^CD4^+^ T cells |  |  | 659 |  |  |
|  | CD3^+^CD8^+^ T cells |  |  | 615 |  |  |
|  | CD16^+^ NK cells |  |  | 148 |  |  |
|  | CD19^+^ B cells |  |  | 1369 |  |  |
|  | Naïve CD19^+^CD27^-^ cells |  |  | 69.3 of CD19^+^ (33-60.7) |  |  |
|  | IgM memory CD27^+^IgM^+^ cells |  |  | **2.6** of CD19^+^ (5.3-22.9) |  |  |
|  | Switched memory CD27^+^IgM^-^ cells |  |  | **0.5** of CD19^+^ (2.5-16.3) |  |  |
| **P7** | Total Lymphocytes |  |  |  | 1428 |  |
|  | CD3^+^ T cells |  |  |  | 1082 |  |
|  | CD3^+^CD4^+^ T cells |  |  |  | 539 |  |
|  | CD3^+^CD8^+^ T cells |  |  |  | 389 |  |
|  | CD16^+^ NK cells |  |  |  | 171 |  |
|  | CD19^+^ B cells |  |  |  | **152** |  |
|  | Naïve CD19^+^CD27^-^ cells |  |  |  | 46.9 of CD19^+^ (33.8-79.6) |  |
|  | IgM memory CD27^+^IgM^+^ cells |  |  |  | 6 of CD19^+^ (3.5-24.1) |  |
|  | Switched memory CD27^+^IgM^-^ cells |  |  |  | 12.6 of CD19^+^ (2.7-20.6) |  |
| **P8** | Total Lymphocytes |  |  |  |  | 1920 |
|  | CD3^+^ T cells |  |  |  |  | 1400 |
|  | CD3^+^CD4^+^ T cells |  |  |  |  | 672 |
|  | CD3^+^CD8^+^ T cells |  |  |  |  | 442 |
|  | CD16^+^ NK cells |  |  |  |  | 110 |
|  | CD19^+^ B cells |  |  |  |  | 350 |
|  | IgM memory CD27^+^IgM^+^ cells |  |  |  |  | **8** of CD19^+^ (8.2-30) |
|  | Switched memory CD27^+^IgM^-^ cells |  |  |  |  | **5.7** of CD19^+^ (7.2-26.3) |
| **P9** | Total Lymphocytes |  |  | 3000 |  |  |
|  | CD3+ T cells |  |  | 2055 |  |  |
|  | CD3+CD4+ T cells |  |  | 1098 |  |  |
|  | CD3+CD8+ T cells |  |  | 528 |  |  |
|  | CD16+ NK cells |  |  | 321 |  |  |
|  | CD19+ B cells |  |  | 249 |  |  |

Normal values (/mmc) are extrapolated from healthy controls of the database of our Laboratory. Total lymphocytes: 6-12 mo 3800-9900; 2-5 yr 1700-6900; 5-10 yr 1100-5900; 10-18 yr 1000-5300; >18 yr 1000-2800. CD3^+^ T cells: 6-12 mo 2400-6900; 2-5 yr 900-4500; 5-10 yr 700-4200; 10-18 yr 800-3500; >18 yr 700-2100. CD3^+^CD4^+^ T cells: 6-12 mo 1400-5100; 2-5 yr 500-2400; 5-10 yr 300-2000; 10-18 yr 400-2100; >18 yr 300-1400. CD3^+^CD8^+^ T cells: 6-12 mo 600-2200; 2-5 yr 300-1600; 5-10 yr 300-1800; 10-18 yr 200-1200; >18 yr 200-900. CD16+ NK cells : 6-12 mo 200-1200; 2-5 yr 100-1400; 5-10 yr 90-900; 10-18 yr 70-1200; >18 yr 90-600. CD19+ B cells: 6-12 mo 700-5200; 2-5 yr 200-2100; 5-10 yr 200-1600; 10-18 yr 200-600; >18 yr 100-500.
