# Supplementary figures and images for: Clinical and immunological data of nine patients with chronic mucocutaneous candidiasis disease
Source: Data Brief. 2016 Feb 23;7:311–5. doi: 10.1016/j.dib.2016.02.040 (PMC4777981; doi:10.1016/j.dib.2016.02.040)

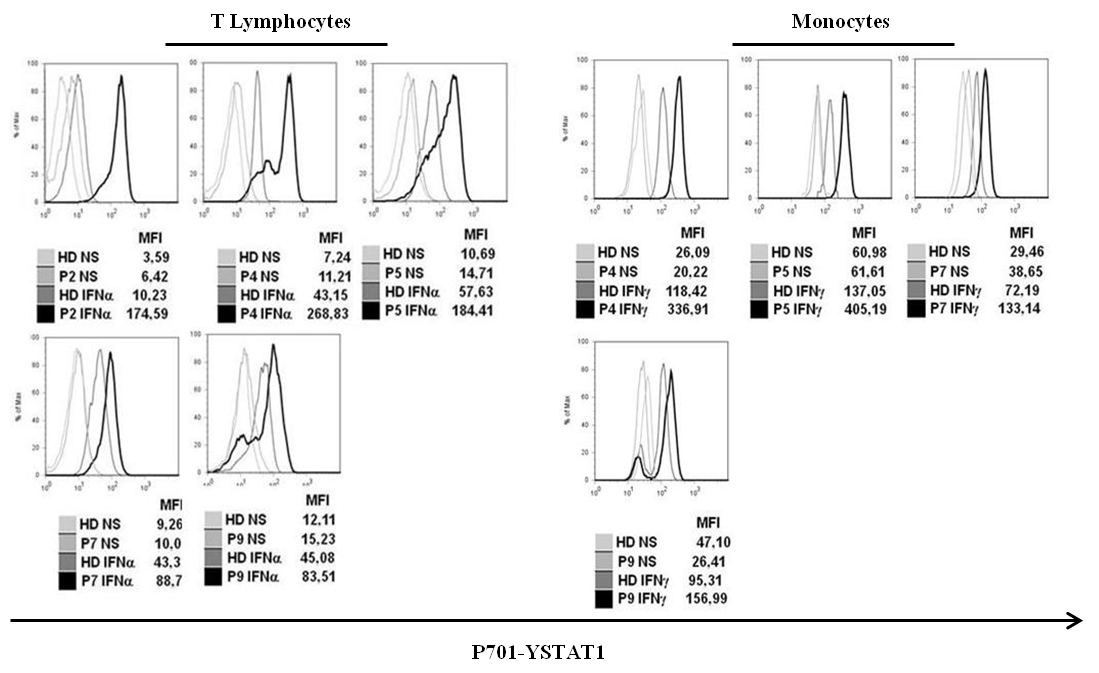

Supplement: Fig 1 — Supplementary material . Intracellular staining of phosphorylated tyrosine 701 STAT1 (P701-YSTAT1) in not stimulated and IFNα-induced T lymphocytes (A), and not stimulated and IFNγ-induced monocytes (B). NS=Not Stimulated; HD=Healthy Donor. Fig 2. Chest Computed Tomography scans showing diffuse bronchiectasis in four GOF-STAT1 patients developing progressing chronic lung disease. [file mmc1.zip › Supplementary Figure 1.tif]

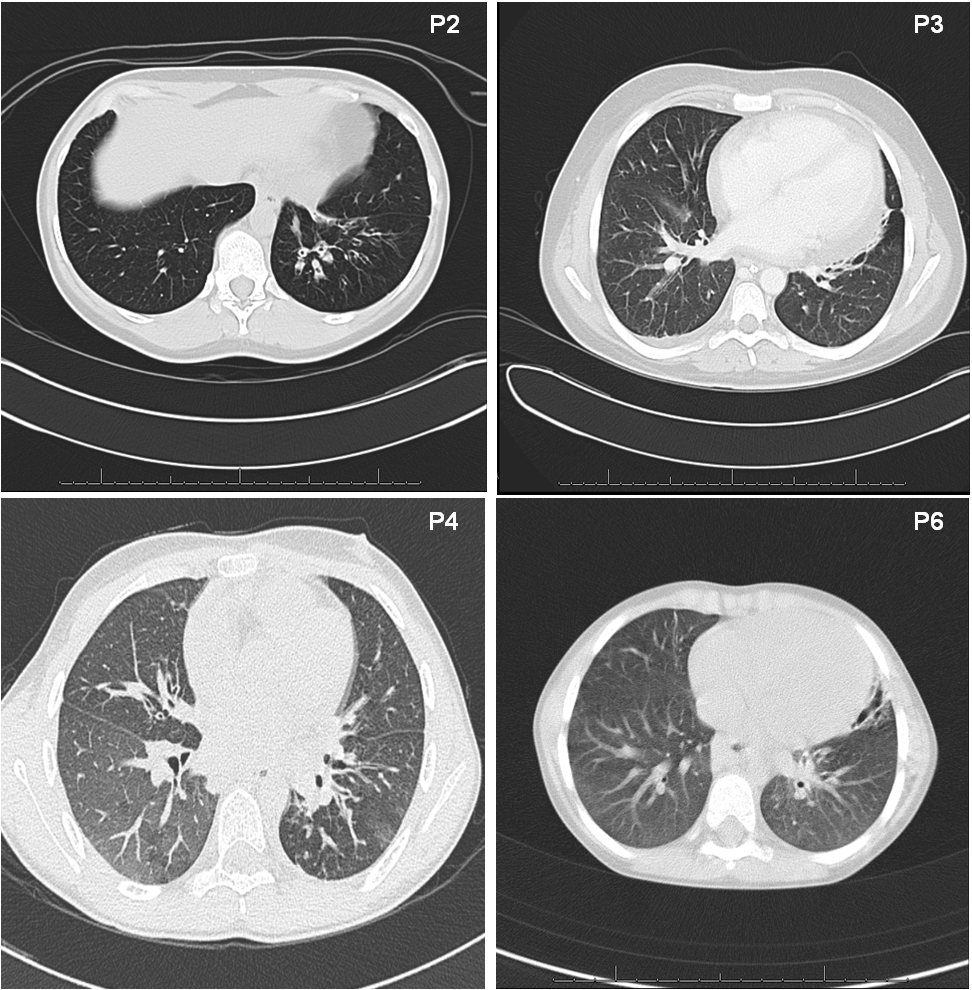

Supplement: Fig 1 — Supplementary material . Intracellular staining of phosphorylated tyrosine 701 STAT1 (P701-YSTAT1) in not stimulated and IFNα-induced T lymphocytes (A), and not stimulated and IFNγ-induced monocytes (B). NS=Not Stimulated; HD=Healthy Donor. Fig 2. Chest Computed Tomography scans showing diffuse bronchiectasis in four GOF-STAT1 patients developing progressing chronic lung disease. [file mmc1.zip › Supplementary Figure 2.tif]
